# Distribution of estrogen and progesterone receptors isoforms in endometrial cancer

**DOI:** 10.1186/1746-1596-9-77

**Published:** 2014-03-31

**Authors:** Hila Kreizman-Shefer, Jana Pricop, Shlomit Goldman, Irit Elmalah, Eliezer Shalev

**Affiliations:** 1 Emek Cancer Diagnosis and Research Institute (ECDRI), Emek Medical Center, Afula, Israel; 2 Laboratory for Research in Reproductive Sciences and Department of Obstetrics and Gynecology, Emek Medical Center, Afula, Israel; 3 Rappaport Faculty of Medicine, Technion-Israel Institute of Technology, Haifa, Israel

**Keywords:** Endometrial carcinoma, Estrogen receptor, Progesterone receptor

## Abstract

**Background:**

70–80% of sporadic endometrial carcinomas are defined as endometrioid carcinoma (EC). Early-stage, well differentiated endometrial carcinomas usually retain expression of estrogen and progesterone receptors (ER and PR, respectively), as advanced stage, poorly differentiated tumors often lack one or both of these receptors. Well-described EC prognosis includes tumor characteristics, such as depth of myometrial invasion. Therefore, in the current study, we evaluated the expression profile of ER and PR isoforms, including ER-α, PR-A and PR–B, in correlation to EC tumor histological depth.

**Methods:**

Using immunohistochemistry and image analysis software, the expression of ER-α, PR-A, PR–B and Ki67 was assessed in endometrial stroma and epithelial glands of superficial, deep and extra-tumoral sections of 15 paraffin embedded EC specimens, and compared to 5 biopsies of non-malignant endometrium.

**Results:**

Expression of PR-A and ER-α was found to be lower in EC compared to nonmalignant tissue, as the stromal expression was dramatically reduced compared to epithelial cells. Expression ratios of both receptors were significantly high in superficial and deep portions of EC; in non-tumoral portion of EC were close to the ratios of nonmalignant endometrium. PR-B expression was low in epithelial glands of EC superficial and deep portions, and high in the extra-tumoral region. Elevated PR-B expression was found in stroma of EC, as well.

**Conclusions:**

The ratio of ER-α and PR-A expression in the epithelial glands and the stroma of EC biopsies may serve as an additional parameter in the histological evaluation of EC tumor.

**Virtual slides:**

The virtual slide(s) for this article can be found here: http://www.diagnosticpathology.diagnomx.eu/vs/1155060506119016

## Introduction

Approximately 70–80% of sporadic endometrial carcinomas are distinguished as type I carcinomas, which is the most common malignancy in female reproductive tract and defined as EC. Well-described EC prognosis includes stage of disease at the time of diagnosis, histologic type, degree of tumor differentiation, depth of myometrial invasion and lymphovascular space invasion. The glandular epithelium from which the cancer arises is hormone responsive, expressing both PRs and ERs [1]. EC often develops from endometrial hyperplasia, which is attributed to prolonged exposure to estrogen in the absence of (unopposed) sufficient progesterone [2], and is often well differentiated and noninvasive or superficially myoinvasive, rarely producing metastases and expressing ER [3]. Whereas early-stage, well differentiated EC usually retain expression of both receptors, advanced stage, poorly differentiated tumors often lack one or both of these receptors, which has been correlated in many studies with a poor prognosis [4,5]. The majority of estrogen-dependent carcinomas occur during the post-menopausal period, when active sex steroids are not produced by the ovaries. Therefore, in-situ estrogen metabolism has a crucial role in the development and progression of EC in this period [6]. Both estrogen and progesterone exert their effect through intra-and extra-nuclear receptors. ER exists in 2 main forms, ER-α and ER-β, encoded by separate genes, *ESR1* and *ESR2* respectively, binding the same estrogen response elements (EREs) and regulate similar sets of genes [7]. However, ER-α and ER-β has a distinct pattern of expression in the tissues [8], which varies during cellular proliferation and differentiation [9]. ER-α is required for the basic development of estrogen sensitive tissues and ER-β is required for organization and adhesion of epithelial cells and hence for differentiated tissue morphology and its functional maturation [10].

PR has been implicated in the development of endometrial cancer, as well. The single-copy PR gene uses separate promoters and translational start sites to produce two isoforms, PR-A and -B [11], which are in fact two functionally distinct transcription factors [12], mediating their own response genes and physiological effects with little overlap [13]. The physiological roles of progesterone in the regulation of endometrial tissue are, in general, considered to antagonize estrogen-mediated cell proliferation and to induce cellular differentiation [14,15]. Loss of total PR expression was found in well and in poorly differentiated EC, and was related to PR-A [16-18]. Highly malignant forms of endometrial, cervical and ovarian cancers have been correlated with over-expression of PR-B [19,20]. Another examined marker in this study is Ki67, a widely used nuclear marker expressed during all active phases of the cell cycle, but absent from resting cells (G0) [21], and therefore its expression is examined in order to assess proliferative activity. High Ki-67 expression was found in various types of endometrial carcinomas [22] and correlates with histological grade, depth of myometrial invasion and risk of recurrence [23-25]. In the current study, the common examination of receptors profile in the epithelial cells of the tumor was under focus by the evaluation of ER and PR isoforms expression as well as Ki67 in the stromal cells and the epithelial glands of EC specimens. Profile of expression was correlated to the tumor histological depth.

## Methods

### Samples collection

15 formalin fixed paraffin-embedded (FFPE) tumor samples from patients diagnosed with grade 1 and 2 EC between March 2007 and February 2010 were obtained from patients undergoing surgery for hysterectomy in the Gynecology department at Emek Medical Center (Afula, Israel). The clinical stage, histological type and tumor grade were assessed using the Federation of Gynecology and Obstetrics (FIGO; 2009) system of classification. The mean age of the patients was 66.2 years with a range from 43 to 87 years. Data of patients is detailed in Additional file 1. Superficial (block 1) and deep (block 2) portions of the tumor, as well as extra-tumoral tissue (block 3) origin in the same specimen, were examined. While the superficial portion represents the surface of the tumor, the deep portion (block 2) represents the myometrial invasion of the tumor, which is an important parameter for the tumor’s characterization, prognosis and adapted treatment. 5 FFPE samples of nonmalignant (normal) endometrial tissue were obtained in the same procedure. Biopsies were numbered, diagnosed and stored in the Emek Cancer Diagnosis and Research Institute (ECDRI). The study was approved by the local ethical committee, Emek Medical Center (Institutional ethical board).

### Tissue processing

Tissues were fixed in 10% paraformaldehyde, processed routinely and embedded in paraffin. Sections (2 μm) were mounted on superfrost slides. Hematoxylin/eosin staining was used for histological evaluation under light microscope. Sequential sections were used for ER-α, PR-A, PR-B and Ki67 stainings.

### Immunohistochemistry

The immunostains were performed on an automated stainer (XT; Ventana Systems, Phoenix, AZ). The primary antibody incubation time for all assays was 32 minutes after antigen retrieval in Tris based buffer (60 minutes at 95–100°C). Anti-ER-α antibody clone H-184 (sc-7207, Santa-Cruz), anti-PR antibody clone 16 (NCL-PGR-312, Novocastra), anti-PR-B antibody clone B-30 (sc-811, Santa-Cruz) and anti-Ki67 antibody clone ZB11 (18-0192Z, Invitrogen) were used. The detection reaction used the iVIEW DAB detection kit (manufacturer-recommended protocol). Hematoxylin counterstain was used for color development.

### Expression of ER-α, PR-A and PR-B using image analysis

Expression assessment of ER-α, PR-A and Ki67 was performed by scoring based on the percentage of stained cells and the intensity of nuclear stain, according to the method described by Carcangiu et al. [26]. Pictures of sections mounted for ER-α, PR-A and PR-B were taken using DP70 Olympus camera. The expression level in the stroma and in the epithelial glands of endometrial tissues was evaluated and compared (Epithelial glands/ Stroma), using the image analysis software Image-Pro Plus (version 4.5.1 for Windows 98/2000/XP/NT 4.0, Media Cybernetics Inc., Bethesda, MD, USA). The epithelial glands/stroma values were examined as a reference tool that presents the relative expression in both glands and stroma cells.

### Statistical analysis

The data was expressed as mean ± standard deviation of mean (SD). Differences in the parameters were evaluated by *t*-test. A *p*-value of less than 0.05 was considered to be significant.

## Results

### ER-α, PR-A and Ki67 scoring

Scoring levels of ER-α, PR-A and Ki67, shown in Table 1 and in Table 2, reflects the common assessment of markers expression, which includes counting of stained epithelial cells detected in 10 high power fields (X40). Average of stained cells is represented by percents. Scoring shows lower expression of ER-α and PR-A in most EC biopsies in both superficial (ER-α 71.7 ± 25.6; PR-A 74.7 ± 29.0) and deep (ER-α 64.7 ± 29.2; PR-A 71.7 ± 29.3) portions, while most extra-tumoral biopsies retains the expression level observed in the nonmalignant tissues (ER-α 90.7 ± 18.3; PR-A 93.7 ± 13.9 in extra-tumoral portion of EC). The expression assessment of Ki67 in EC is also aberrant, and was found to be higher in most superficial EC biopsies (45.7 ± 15.7) than in nonmalignant endometrial specimens.

**Table 1:** Scoring of ER-α, PR-A and Ki67 in epithelium of superficial, deep and extra-tumoral sections of EC

	**ER-α (mean of % positive cells)**			**PR-A (mean of % positive cells)**			**Ki67 (mean of % positive cells)**		
	**Superficial**	**Deep**	**Extra-tumoral**	**Superficial**	**Deep**	**Extra-tumoral**	**Superficial**	**Deep**	**Extra-tumoral**
Mean (± SD)	71.7(±25.6)	64.7(±29.2)	90.7(±18.3)	74.7(±29.0)	71.7(±29.3)	93.7(±13.9)	45.7(±15.7)	28.3(±15.7)	2.3(±2.2)
Range	20-100	0-100	50-100	20-100	0-100	50-100	10-70	5-50	<1-10

**Table 2:** Scoring of ER-α, PR-A and Ki67 in epithelium of non-malignant endometrial specimens

**Patient num.**	**ER-α (mean of % positive cells)**	**PR-A (mean of % positive cells)**	**Ki67 (mean of % positive cells)**
08-23394	100	100	50
08-27832	100	100	<1
08-26747	80	100	25
08-28158	100	100	50
08-27037	40	0	<1

### Expression of ER-α in EC

**Figure 1:**
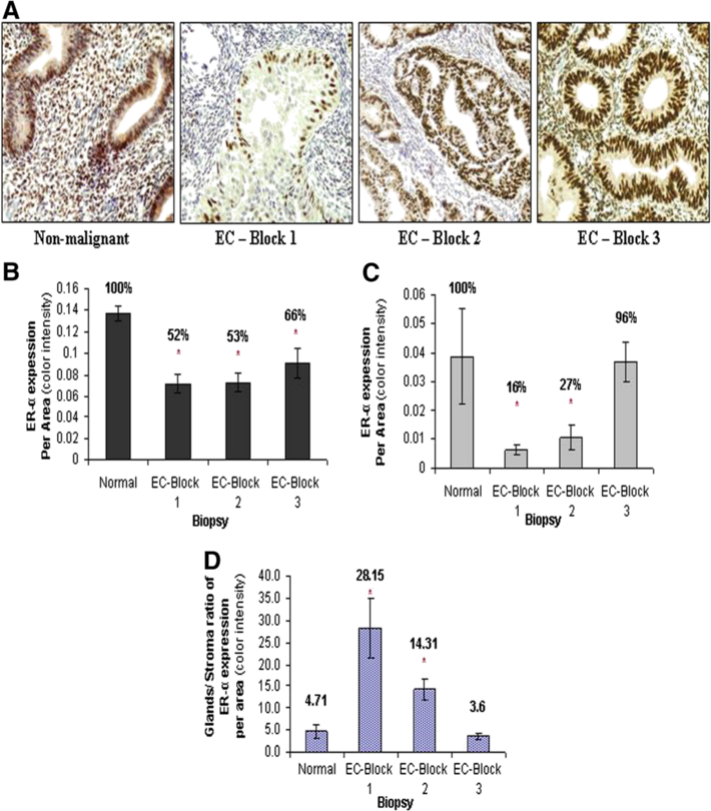
**Expression of ER-α in EC.** Representative sections of the superficial (Block 1), deep (Block 2) and extra-tumoral portion (Block 3) of EC were stained with anti-ER-α antibody and compared with stained sections of nonmalignant endometrial specimens (Normal), as seen in the photographs **(A)**. The expression level was examined in epithelial cells **(B)**, stroma cells **(C)** and the relative expression of both cell types (epithel/ stroma) **(D)** was calculated. Asterisks mark statistical significance (P < 0.05) compared to nonmalignant endometrial tissue (normal) (X400).

Results show reduced ER-α expression in all portions of EC (Figure 1). Superficial portion was found to be affected the most, as well as the stroma cells of all portions. Expression ratio (Glands/stroma) in the surface of EC specimens was found to be significantly higher (28.15 ± 6.72) than in the nonmalignant specimens (4.71 ± 1.51). The ratio is lower in the deep portion of the tumor (14.31 ± 2.52). Extra-tumoral portion shows a ratio close to the nonmalignant tissue (3.6 ± 0.66).

### Expression of PR-A in EC

**Figure 2:**
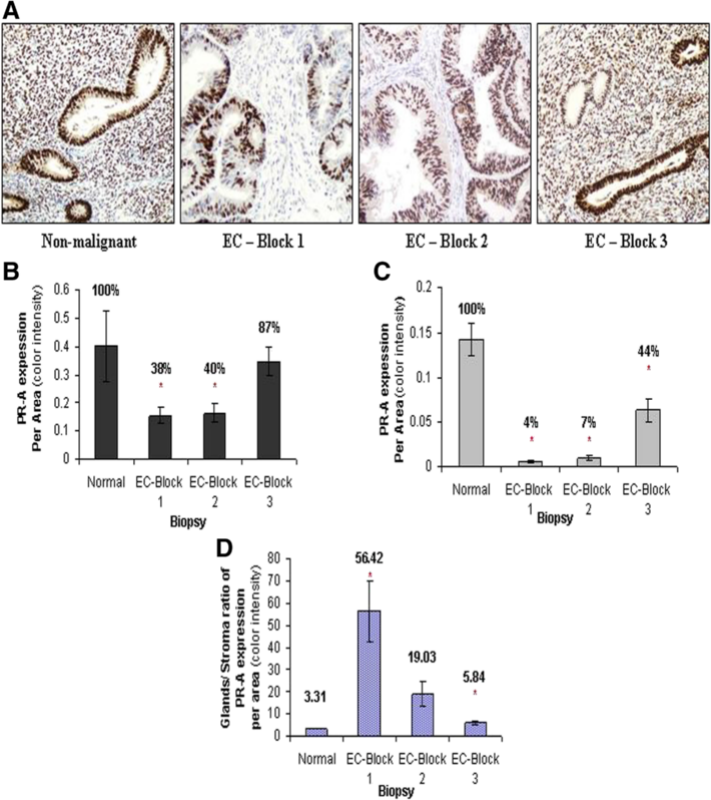
**Expression of PR-A in EC.** Representative sections of the superficial (Block 1), deep (Block 2) and extra-tumoral(Block 3) portions of EC, as well as nonmalignant endometrial specimens (Normal) were examined for PR-A expression, as seen in the photographs **(A)**. The expression level was examined in epithelial cells **(B)**, stroma cells **(C)** and the relative expression of both cell types (epithel/stroma) **(D)** was calculated. Asterisks mark statistical significance (P < 0.05) compared to nonmalignant endometrial tissue (normal) (X200).

PR-A expression was previously found to be highly correlated with the expression of ER [27]. Our results, shown in Figure 2, are supportive of this postulation, as the pattern of PR-A expression shows the same trend in the different portions of EC specimens as ER-α, as well as the ratio of expression (Glands/stroma) (Superficial 56.42 ± 13.55; Deep 19.03 ± 5.43; Extra-tumoral 5.84 ± 0.9).

### Expression of PR-B in EC

**Figure 3:**
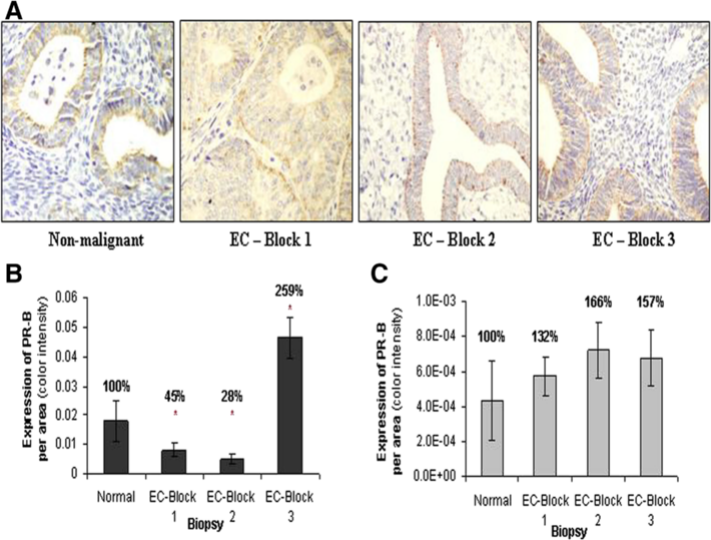
**Expression of PR-B in EC.** PR-B expression was assessed in the superficial (Block 1), deep (Block 2) and extra-tumoral (Block 3) portions of EC, as well as nonmalignant endometrial specimens (normal), as seen in the photographs **(A)**. The expression level was examined in epithelial cells **(B)** and in the stroma cells **(C)**. Asterisks mark statistical significance (P < 0.05) compared to nonmalignant endometrial tissue (normal) (X400).

Whereas non-ligated ER-α and PR-A are localized predominantly in the nucleus, PR-B is often cytoplasmic as well as nuclear (Figure 3) [28,29]; therefore, its detection was more complex, and precision was harder to achieve. In this state, the calculation of expression ratio was not informative. In the epithelial glands PR-B showed a diverse expression, and was found to be higher in the stroma cells of all EC portions (Superficial 132% ± 25%; Deep 166% ± 36%; Extra-tumoral 157% ± 36%).

## Discussion

Molecular tumor classification, which includes PR and ER expression, is an integral part of the disease characteristics. The presence of steroid receptors ER-α, PR-A and PR-B has been quantitatively associated with histologic differentiation [30,31], response to therapy [32] and metastatic potential [33]. ER-α expression was found to be decreased in EC [18,34] and is further decreased as EC grading is advanced [35-38]. In correlation, our results demonstrate significantly reduced expression of ER-α in both glands and stroma of endometrioid tumor in relation to non-malignant endometrial tissue (Figure 1). The expression of ER-α is lower in the stroma than in the glands of EC, indicating that stroma cells are significantly more affected than the epithelial cells. ER-β quantification faced technical problems and therefore was not assessed in the current study. Loss of ER suggests an advanced molecular pathology of the tumor with the deregulation of signaling pathways. Common deregulation courses include PTEN inactivation by mutation [39], *de novo* methylation of ER-α gene and aberrant methylation of CpG islands [1]. These epigenetic alterations occur in a wide variety of tumors [8,40-44], including endometrial cancer [36,45].

PR expression of either one or both of the two PR isoforms was found to be reduced or absent in endometrial cancer [16-18,46], mostly lower for the higher histological grade [47-49] and inversely correlates with myometrium invasion [50,51]. Our results demonstrate that PR-A shows the exact same pattern of expression as ER-α in the gland and stroma cells, as well as in the different portions of EC specimens (Figure 2). It is well documented in the literature that the transcription of *PR* gene is induced by estrogen and inhibited by progesterone in the majority of estrogen responsive cells, so the expression of ER and PR is considered to be coordinated [27,37,52,53]. As described, we found significantly and differentially altered expression of sex steroid receptors in superficial and deep sections of the specimens. Previous reports, which support our findings, describe total protein expression in the tissue. Our findings, describing the expression of PR-A and ER-α in the stroma and epitheial cells in EC solely, is implicated in the mitogenic response of epithelial cells to estrogen, which is mediated indirectly by stromal ER [54]. A model for this assumption was demonstrated by co-culture of non-expressing ER stroma cells and ER-positive epithelial cells [55]. No epithelial proliferation in response to estrogen was detected in this model, or in a model of pure epithelial cultures, an induction observed in co-cultures of normal uterine stroma and epithelial cells [55]. Evidently estrogen induced epithelial proliferation requires an ER-positive stroma. Response of uterine epithelial cells to progesterone was also found to be mediated by stromal PR [56]. This mediated operation between the cells may be implicated and result in the altered pattern of receptors expression in the transformed cells, found in our study. In addition to the well-known growth inhibiting effect of progesterone, it plays an important role in regulating invasive properties of endometrial cancer cells. A correlation was found between decreased PR expression in EC tumors and the expression of E-cadherin and myometrial invasion [57,58]. An extensive myometrial invasion may be a progeny of epithelial to mesenchymal transition (EMT) which is highly implicated in EC tumors invasive characteristics [59,60]. Therefore, the reduced expression of ER-α and PR-A in the tumor cells, particularly the significantly reduced expression in the stroma cells, may indicate an invasive characteristics of the tumor, as described for ER-α [61], and the deep portion of the tumor is of special interest. These findings in both superficial and deep portions of the tumor stand against the extra-tumoral portion of the tumor, which was found to be affected as well, but to a lower extent. PR-B quantification showed reduced expression in the epithelial glands of superficial and deep portions of EC (Figure 3). Supporting our findings, PR-B promoter was previously found to be methylated in endometrial carcinoma [62] and the loss of expression was referred to as an independent prognostic factor for cause-specific survival in high risk patients [63]. The significantly high expression of PR-B in the extra-tumoral portion of the malignant specimens may imply a certain protective reaction opposing invasive properties of the tumor cells. In a study conducted by Balmer NN et al. [64], in which tumoral- and extra-tumoral- portions were examined by immunohistochemistry, resembling the current study methodology, PR-B expression was found to be significantly higher in carcinoma-associated nonmalignant endometrium compared to endometrial carcinoma. Zafran et al. [65] found that a state of PR-B dominance, like in the cell line HEC-1A, was less invasive than cell lines that PR-A is the predominantly expressed variant. PR-A may be associated with a cell- and promoter specific repression of PR-B [66] and imbalance in PR-A to PR-B ratio is frequently associated with carcinogenesis [67]. The relative over-expression of PR-B, which is referred to as an endometrial estrogen agonist [68], without transcriptional repression by PR-A, as shown in our findings, may also be related to the metastatic potential and partially cause deviation from sex steroidal dependency in endometrial cancers [33]. Our results show higher expression of Ki-67 in the malignant tissue than in the nonmalignant, as seen in previous studies [22-25,69]. A wide score range of Ki67 expression was found in the non-malignant biopsies. These results correlate with the expression of Ki67 in normal cyclical endometrium, in which Ki-67 staining is intense and diffused in the proliferative phase, but decreases dramatically in the early and mid-secretory phase.

## Conclusions

In the current study, we have showed the importance of referring to steroid receptors profile in the stroma as well as the epithelial cells. The problem in attaining a consensus regarding assessment of endometrial carcinomas was recently discussed [70,71] and updating the pathologist biomarkers panel was shown to be useful in characterizing EC tumors and in patients prognosis [72-74]. Studies of steroid receptors pattern of expression help in understanding their mechanism of action in target tissues, and could be helpful in defining biologically different subgroups and therapeutic efficacy. We have found that the ratio of ER-α and PR-A expressions in the epithelial glands and the stroma of EC biopsies has a distinct values in different portions of the tumor. These findings may serve in the marker panels of the pathologist in order to improve diagnostic reproducibility. It should be noted that this study has focused on a small and limited group of biopsies. Further analysis in large scale study may contribute to the understanding of ER and PR isoforms expression in EC, and a possible use of ER-α and PR-A relative expression as a clinical tool.

## Abbreviations

EC: Endometrioid carcinoma; ER: Estrogen receptor; PR: Progesterone receptor; EREs: Estrogen response elements; FFPE: Formalin fixed paraffin-embedded; EMT: Epithelial to mesenchymal transition.

## Competing interests

The authors declare that they have no competing interests.

## Authors’ contributions

ES, IE and SG participated in the study design, study analysis and manuscript reviewing. JP participated in conducting the study. HKS participated in conducting the study, study analysis and manuscript writing. All authors read and approved the final manuscript.

## Supplementary Material

Additional file 1 Patients epidemiological and pathological data. Click here for file
