# Supplementary material for: Distribution of estrogen and progesterone receptors isoforms in endometrial cancer
Source: Diagn Pathol. 2014 Mar 31;9:77. doi: 10.1186/1746-1596-9-77 (PMC4022268; doi:10.1186/1746-1596-9-77)
Supplement: Additional file 1 — Patients epidemiological and pathological data. [file 1746-1596-9-77-S1.docx]

Table 1: Patients epidemiological and pathological data.

|  | Patient Num. | Date of Surgery | Age | Grade | Stage |
| --- | --- | --- | --- | --- | --- |
| EC Biopsies | 07-5942 | 12/03/2007 | 73 | 2 | 1A |
|  | 08-8622 | 10/04/2008 | 57 | 2 | 1A |
|  | 08-9200 | 17/04/2008 | 70 | 1 | 1B |
|  | 08-12627 | 05/06/2008 | 74 | 1 | 1A |
|  | 08-14238 | 26/06/2008 | 67 | 1 | 1A |
|  | 08-22044 | 25/09/2008 | 76 | 2 | 2 |
|  | 08-22862 | 15/10/2008 | 69 | 1 | 2 |
|  | 08-26273 | 26/11/2008 | 73 | 1 | 2 |
|  | 09-5662 | 09/03/2009 | 87 | 1 | 1B |
|  | 09-8225 | 07/04/2009 | 70 | 1 | 1A |
|  | 09-18223 | 11/08/2009 | 77 | 2 | 4B |
|  | 09-23021 | 19/10/2009 | 76 | 1 | 1B |
|  | 09-23472 | 22/10/2009 | 72 | 1 | 1A |
|  | 10-2719 | 01/02/2010 | 55 | 1 | 1A |
|  | 10-2767 | 01/02/2010 | 59 | 2 | 2 |
| Non- malignant Biopsies | 08-23394 | 27/10/2008 | 43 |  |  |
|  | 08-26747 | 04/12/2008 | 47 |  |  |
|  | 08-27037 | 09/12/2008 | 45 |  |  |
|  | 08-27832 | 18/12/2008 | 85 |  |  |
|  | 08-28158 | 22/12/2008 | 49 |  |  |
